# Salivary Hormones Response to Preparation and Pre-competitive Training of World-class Level Athletes

**DOI:** 10.3389/fphys.2015.00333

**Published:** 2015-11-16

**Authors:** Gaël Guilhem, Christine Hanon, Nicolas Gendreau, Dominique Bonneau, Arnaud Guével, Mounir Chennaoui

**Affiliations:** ^1^Laboratory Sport, Expertise and Performance (EA 7370), Research Department, French National Institute of Sport (INSEP)Paris, France; ^2^Fatigue and Vigilance Unit (EA 7330), Neurosciences and Operational Constraints Department, French Armed Forces Biomedical Research Institute (IRBA), Paris Descartes UniversityBrétigny-sur-Orge, France; ^3^Laboratory “Movement, Interactions, Performance” (EA 4334), University of NantesNantes, France

**Keywords:** alpha-amylase, immunoglobulin A, chromogranin A, creatine kinase, athletics training

## Abstract

This study aimed to compare the response of salivary hormones of track and field athletes induced by preparation and pre-competitive training periods in an attempt to comment on the physiological effects consistent with the responses of each of the proteins measured. Salivary testosterone, cortisol, alpha-amylase, immunoglobulin A (IgA), chromogranin A, blood creatine kinase activity, and profile of mood state were assessed at rest in 24 world-class level athletes during preparation (3 times in 3 months) and pre-competitive (5 times in 5 weeks) training periods. Total mood disturbance and fatigue perception were reduced, while IgA (+61%) and creatine kinase activity (+43%) increased, and chromogranin A decreased (−27%) during pre-competitive compared to preparation period. A significant increase in salivary testosterone (+9 to +15%) and a decrease in testosterone/cortisol ratio were associated with a progressive reduction in training load during pre-competitive period (*P* < 0.05). None of the psycho-physiological parameters were significantly correlated to training load during the pre-competitive period. Results showed a lower adrenocortical response and autonomic activity, and an improvement of immunity status, in response to the reduction in training load and fatigue, without significant correlations of salivary hormones with training load. Our findings suggest that saliva composition is sensitive to training contents (season period) but could not be related to workload resulting from track and field athletics training.

## Introduction

During the last few decades, the exponential increase in international competition has progressively led the top-level athletes to extend the time spent to train in order to enhance their performance. Although successful training must involve such high training load phases (i.e., increased volume and intensity), it must also avoid the combination of excessive overload and inadequate recovery (Meeusen et al., 2006). Indeed, a disrupted balance between training stress and rest period following exercise can increase fatigue associated with short-term withdrawal of performance capacity, defined as functional “overreaching” syndrome (Meeusen et al., 2006). When intensive training is maintained, overreaching might evolve to a non-functional overreaching state determined by “qualitative” changes (i.e., symptoms of psychological and/or endocrine distress), which necessitate several weeks or months to restore initial performance (Meeusen et al., 2006). In a monitoring training study, Foster (1998) noted a correspondence between excessive training load and injury and illness occurrence, thereby highlighting the interest of methods for the identification of non-functional overreaching development in elite athletes. Although a very large panel of variables exist to detect non-functional overreaching, on the one hand, there is a lack of consensus regarding their significance when considered independently (Meeusen et al., 2006). On the other hand, multivariate approaches have shown promising results (Le Meur et al., 2013), but have to be validated in the natural context of training and may consequently have limited practicality when used in field conditions of high-level training.

Physical activity is an external stimulus able to trigger an adaptive response coordinated by the stress system (Chennaoui et al., 2004). The main components of the stress system include the autonomic nervous system (ANS) and the hypothalamic-pituitary adreno-cortical (HPA) axis, whose function is sensitive to psychological and physiological stressors (Tsigos and Chrousos, 2002). The ANS mediates the stress response via parasympathetic and sympathetic nerves to the adrenal medulla to produce catecholamines, while steroid hormones are the final effectors of HPA. Thereby, the plasma concentrations of catecholamines, or corticosteroids, testosterone (T) and cortisol (C), have been used as an indicator of autonomic activity (Urhausen et al., 1995). However, the quantification of these proteins also require invasive, stressful procedures (sting venipuncture) and qualified personnel to collect blood samples. Released in blood circulation, steroid hormones may also reach saliva by passive diffusion or active transport.

Scientific evidence also suggests that autonomic activity can indirectly be assessed by hormones released in saliva under the control of ANS (Nater and Rohleder, 2009). Several studies reported fatigue-induced variations in salivary testosterone, signaling a catabolic state, in relation to the intensity and duration of a preceding physical load (Gatti and De Palo, 2011), with an effect of age and gender on basal values (250–600 for males vs. 200 pmol.L^−1^ for females) (Wood, 2009). Similarly, salivary cortisol can be used to determine psychophysiological stress during single and repeated exercise sessions even if a non-univocal relationship has been found between stress and cortisol concentration (Gatti and De Palo, 2011). In addition, during physiological stress experience, similar mechanisms underlie the secretion of alpha-amylase (AA), and catecholamines (norepinephrine and epinephrine), which are co-stored and co-released in saliva with chromogranin A (Montero-Hadjadje et al., 2008). Similarly, subjects exposed to excessive stress could experience immunodepression, which is manifested by decreased levels of immunoglobulin A (IgA), secreted in saliva under autonomic control (Papacosta and Nassis, 2011). Interestingly, repeated training sessions have been shown to increase the levels of salivary IgA and AA activity (Born et al., 2015) or decrease the salivary CgA concentration (Díaz Gómez et al., 2013). Taking together, these elements make saliva a useful, non-invasive, rapid and stress-free alternative to the collection of serum and plasma, allowing for frequent and easy appraisal of stress response.

In this context, the impact of training on salivary markers is currently receiving growing attention. Indeed, the response of aforementioned proteins (e.g., T, C, AA, CgA, IgA) has been applied to predict exercise intensity in well-trained athletes with variable outcomes (Gatti and De Palo, 2011). These different findings have been notably attributed to the high inter-individual variability of the salivary biomarkers, suggesting that repeated sampling can provide more accurate results than unique measure (Papacosta and Nassis, 2011). Whereas most of the studies measured response to acute exercise, those reporting association with chronic exercise (long-term training) in humans are scarce (Nater and Rohleder, 2009). Recently, Diaz et al. (2013) showed that salivary AA response was proportional to training load and intensity in swimmers. However, little is known about the potential usefulness of salivary markers in the identification of non-functional OR over prolonged periods, notably in sport activities with high incidence of injury (e.g., team sports, running, jumping).

Thus, the aim of the present study was to determine the long-term changes in endocrine, psychological, and muscle damage response of training stimulus during an injurious period of elite track and field athletics season, in comparison to a normal preparation phase. Preliminary data and previous studies showed that injury incidence increased during the pre-competitive period, where training volume progressively decreases to increase session intensity and focus on technical contents (D'souza, 1994). We hypothesized that such modifications in the training stress influence salivary proteins throughout training. Such quantified changes could help to reduce the risk of injury, adjust volume and intensity of training, and in turn lead to enhance functional gains.

## Methods

### Participants

Twenty-four high-level track and field athletes (9 men, 15 female, 25 ± 4 years; 177 ± 12 cm; 67 ± 12 kg), including short and long distance sprinters (100–400 m), long jumpers, middle distance runners (800–1500) and combined events athletes, all members of the French national team and competing at the international level during the year of the experiment, participated in this study. The sample included two World champions and one Olympic champion, and several medallists at the European championships, all ranked between the 70th and the 1st place at the International Association of Athletics Federations at the moment of the study. All participants were informed regarding the nature, aims, and risks associated with the experimental procedures before they gave their written consent to participate. None of them smoked, had significant medical or oral health history, or were taking regular or incidental medication during the study. The study was approved by the ethics committee of Paris XI and the French health and safety agency. All experiments were conducted in accordance with the Declaration of Helsinki.

### Experimental design

**Figure 1:**
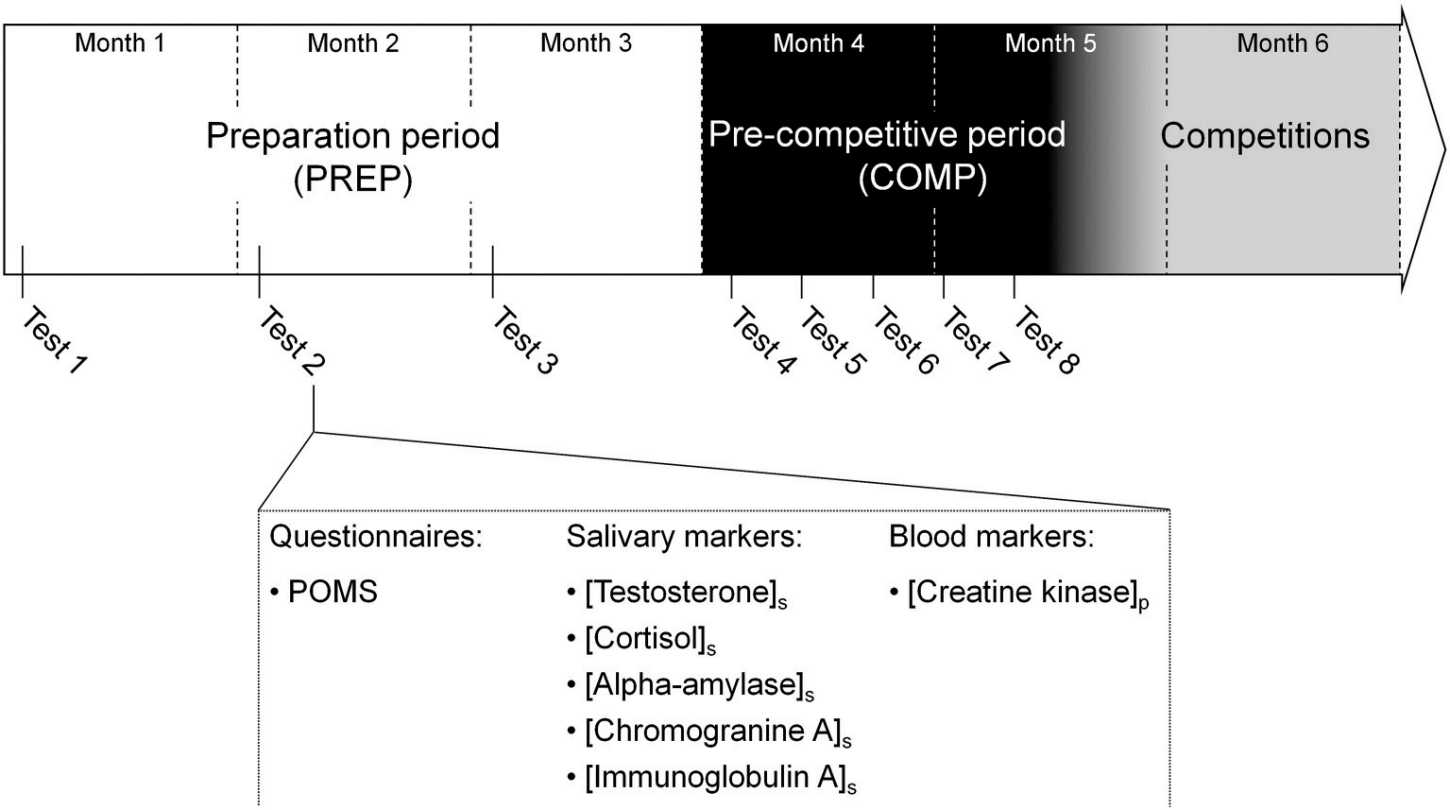
**Overview of the experimental design**. A total of eight tests sessions were spread over the 5 months prior to the international competition period. The first three tests were performed during the first week of 3 month of the preparation period (PREP) consisted of high volume and low intensity contents. During the pre-competitive period (COMP) characterized by low volume and high intensity training, identical tests sessions were performed every Monday during 5 weeks. Each test session included a Profile Of Mood State (POMS) questionnaire, salivary and blood samples to assess total mood disturbance, salivary concentration of testsosterone, cortisol, alpha-amylase, immunoglobulin A and chromogranin A and plasma creatine kinase activity.

All participating athletes were followed throughout a 20 week (i.e., 4.5 months) period prior to the major international competitions (Figure 1). The follow-up was spread over two main periods: a preparation period of 3 months (PREP), characterized by a high training volume, and a pre-competitive period (COMP) of 1.5 month including high-intensity and specific training, with a reduced training volume (Issurin, 2010). This part of the season was previously identified as the period where injury incidence was the highest for the athletes (personal data; D'souza, 1994). During the preparation period, aimed at determining basal hormonal profile, participants attended a test session every first Monday of each month. During pre-competitive period, tests were performed every Monday during 5 consecutive weeks to provide more accurate salivary hormones quantification throughout this phase where injury incidence increases (Papacosta and Nassis, 2011). Each test session consisted of psychological questionnaires filling, and collection of saliva and blood samples. The participants refrained from intense exercise at least 24 h prior to the each collection of samples.

### Data collection

#### Psychometric measurements

The participants completed the Profile Of Mood States (POMS) immediately before saliva collection (Cayrou et al., 2003). The participants were asked to state how they felt over the week. The POMS is a 65-item questionnaire measuring tension, depression, anger, confusion, vigor, and fatigue on a 5-point Likert scale. The internal consistency for the POMS (Cronbach's alpha coefficient) was 0.96.

#### Samples

Whole saliva and blood samples were performed after at least 24 h of rest, the same day of the week, at the same time of the day (4 p.m.) for all athletes to control for diurnal variation (Gatti and De Palo, 2011). T, C, AA, IgA, and CgA were measured from saliva samples, while CK activity was determined from blood sample. According to standard recommendations, the participants were asked to refrain from drinking or eating during the 2 h prior to the collection of the samples. They were instructed not to perform any physical exercise 2 h and not to brush their teeth 45 min prior to sampling to avoid micro-injuries or abrasion that could induce blood contamination of saliva. Athletes washed their mouth with water 10 min before collection and swallowed the first amount of saliva. Whole saliva was collected by passive drool with no exogenous stimulation, recognized as the most reliable option (Granger et al., 1994). The saliva was allowed to pool in the mouth and then drooled into pre-weighted collection vials after 2 min. Immediately after collecting saliva, 5-mL blood samples were collected in vacutainer EDTA-coated tubes via antecubital venipuncture on the resting arm. Tubes were then mixed and placed on ice before centrifugation (10 min at 2000 bpm at 4°C) and plasma was transferred using a pipette into Eppendorf tubes. Tubes containing plasma and saliva were stored at −80°C for further analysis.

#### Training load

During PREP, the training sessions focused on strength and aerobic development with the objective of maintaining the qualities of velocity and the technical abilities. The COMP period consisted of improvement of the anaerobic qualities (velocity and speed endurance) while preserving the retention of the aerobic level. Over this period, the technical exercises were performed at a maximal velocity. Training load was quantified for each week of COMP, in order to relate psychometrical and biological changes to training stimulus throughout the injurious period (i.e., COMP). According to the Foster model, the training load of each athlete was determined for each session by integrating the exercise session rating of perceived exertion (RPE) and the duration of the training session (Foster, 1998).

### Data analyses

Salivary concentrations of testosterone, cortisol, and IgA and AA activity were determined using a spectrophotometer Dynex MRXe (Magellan Biosciences, Chelmsford, USA) and standard assay kits (Salimetrics, State College, PA, USA). Chromogranin A was assayed using a kit from Yanaihara Institute (YK070 Human CgA EIA, Yanaihara Institute, Shizuoka, Japan). Salivary concentrations were determined from duplicates of the samples obtained during the preparation, resulting in an intra-assay coefficient of variation below 5% for all salivary markers (4.5 ± 2.1% on average). Subsequent analyses (pre-competitive phase) were thus performed in simple measurement. Measurements performed during both periods were averaged to obtain a representative value for each period (3 values for preparation period, 6 values for pre-competitive period).

#### Testosterone (T)

The concentration of salivary testosterone in the sample is inversely proportional to the testosterone peroxidase, which was measured by the reaction of the peroxidase enzyme on the substrate tetramethylbenzidine. The optical density resulting from this reaction was measured at 450 nm.

#### Cortisol (C)

The amount of cortisol peroxidase, measured by the intensity of color originate from the reaction of the peroxidase enzyme on the substrate tetramethylbenzidine, is inversely proportional to the amount of cortisol present. The optical density was measured at 450 nm. The ratio between testosterone and cortisol salivary concentration was expressed as the T/C ratio (Adlercreutz et al., 1986).

#### Alpha-amylase (AA)

The reagents in the kit contain a chromagenic substrate, 2-chloro-p-nitrophenol linked with maltotriose. The amount of AA activity present in the sample is directly proportional to the increase in absorbance resulting from the enzymatic action of AA on this substrate yields 2-chloro-p-nitrophenol, which was spectrophotometrically measured at 405 nm. Inter- and intra-assay variance was below 1%.

#### Immunoglobulin A (IgA)

The salivary concentration of IgA was determined by measuring the optical density of the reaction of the peroxidase enzyme on the substrate tetramethylbenzidine, at 450 nm. The amount of peroxidase is inversely proportional to the amount of IgA present in the sample.

#### Chromogranin A (CgA)

The salivary concentration of CgA was determined by competitive enzyme immunoassay using combination of highly specific antibody to human CgA (344–374) and biotin-avidin affinity system. The 96-wells plate was coated with goat anti rabbit IgG. Human CgA standard or samples, labeled antigen and specific antibody, were added to the wells for competitive immunoreaction. After incubation and plate washing, HRP labeled streptoavidin (SA-HRP) were added to form HRP labeled streptoavidin-labeled antigen-specific antibody complex on the surface of the wells. Finally, HRP enzyme activity was determined by o-Phenylenediamine dihydrochloride (OPD) and the concentration of human CgA is calculated by measuring the absorbance at 490 nm.

#### Plasma CK activity

As a marker of sarcolemma disruption, plasma CK activity was measured spectrophotometrically by using commercially available reagents (Roche/Hitachi, Meylan, France) with an inter-assay precision (CV) of 2.7 ± 1.3%.

### Statistical analysis

All analyses were performed with Statistica Version 7.1 (StatSoft, Tulsa, Oklahoma, USA). Data distributions consistently passed the Shapiro-Wilk normality test. All data being normally distributed, Two-way ANOVAs with repeated measures (gender × training period) were performed to determine potential differences in average values of psychometric measurements (POMS scores), salivary concentrations of T, C, AA, IgA, CgA, and blood CK activity between preparation and pre-competitive periods. One-way ANOVAs (time effect) with repeated measures were applied to determine changes in POMS scores, [T], [C], AA activity, [IgA], [CgA], CK activity, and training load throughout the pre-competitive period. A Geisser-Greenhouse correction was used when the sphericity assumption in repeated measures ANOVAs was violated (Mauchly's test). *Post-hoc* tests were performed by means of Newman-Keuls procedures. Separate linear Pearson correlations (r) were performed between each indicators and training load. The significance level was set at *P* < 0.05 for all tests. The data are presented as mean ± SD.

## Results

### Mood disturbance

Tension—anxiety, depression, anger and confusion—bewilderment scores significantly decreased from PREP to COMP (*P* < 0.05), whereas vigor—activity (*P* = 0.4) and fatigue (*P* = 0.07) were not affected between both training periods As a result, total mood disturbance was significantly decreased when competitions approached, in comparison to the PREP (Table 1). All POMS subscale scores and total mood disturbance did not change significantly throughout COMP (*P* = 0.15–0.68; Table 2).

**Table 1:** **Mood disturbance scores of track and field athletes during the preparation (PREP) and pre-competitive (COMP) period of the sport season**.

**POMS subscale scores**	**PREP**	**COMP**
Tension—anxiety	8.6 (4.5)	6.8 (4.0)^*^
Depression	7.6 (9.1)	4.6 (6.2)^*^
Anger—hostility	10.4 (8.2)	7.0 (7.7)^*^
Vigor—activity	16.5 (4.2)	15.8 (4.8)
Fatigue	7.0 (4.2)	5.5 (3.2)
Confusion—bewilderment	7.1 (4.3)	5.1 (3.2)^*^
Total mood disturbance	43.1 (20.4)	33.7 (17.6)^*^

*The results are means (SD)*.

* *Significant difference (P > 0.05)*.

**Table 2:** **Training load and mood disturbance of track and field athletes throughout the 5 weeks of the pre-competitive (COMP) period of the sport season**.

**POMS subscale scores**	**Training week of the pre-competitive period (COMP)**				
	**1**	**2**	**3**	**4**	**5**
Training load	3746.4 (4353.3)	2479.1 (3044.2)	2953.2 (2556.3)	1230.8 (1694.2)	1072.7 (1428.2)^*^
Tension—anxiety	7.7 (4.8)	7.4 (4.4)	7.0 (5.3)	6.6 (5.0)	5.5 (4.8)
Depression	5.1 (6.8)	5.5 (7.9)	4.3 (8.7)	7.5 (10.2)	3.6 (6.0)
Anger—hostility	8.1 (9.0)	6.8 (7.5)	6.6 (9.8)	9.7 (11.0)	6.7 (6.9)
Vigor—activity	16.2 (5.3)	15.2 (5.6)	16.1 (6.5)	16.0 (6.2)	15.0 (6.0)
Fatigue	6.0 (4.7)	6.6 (4.9)	5.0 (4.5)	5.6 (4.3)	4.8 (4.0)
Confusion—bewilderment	6.0 (3.9)	5.8 (4.6)	4.9 (4.3)	4.4 (4.2)	4.3 (4.0)
Total mood disturbance	37.0 (19.9)	35.5 (16.9)	32.6 (24.7)	33.4 (25.4)	31.3 (17.0)

*The results are means (SD)*.

* *Significant effect of time (P > 0.05)*.

### Salivary hormones

#### Testosterone

**Figure 2:**
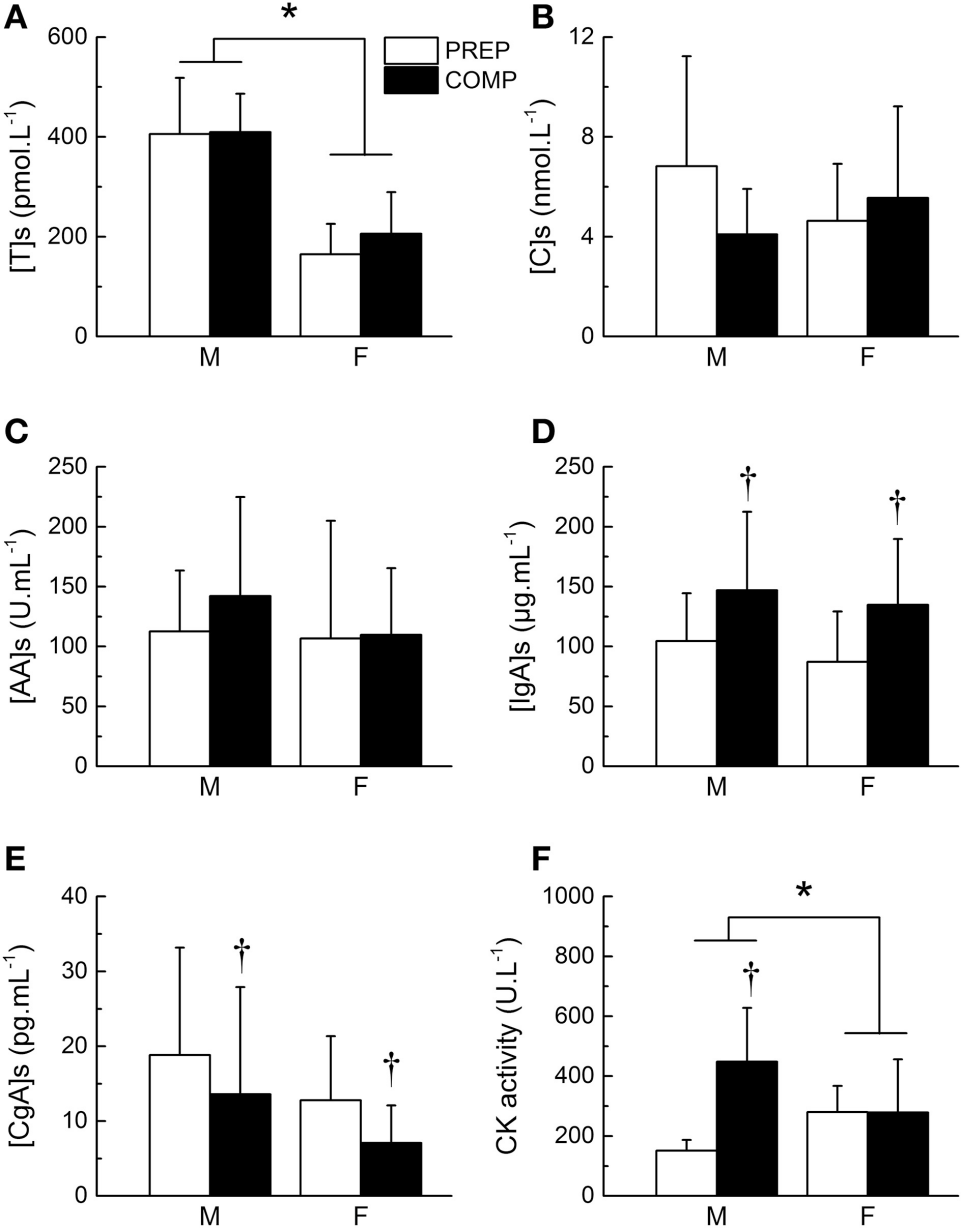
**Changes in biological markers between preparation and competitive training periods**. Mean salivary concentration of testosterone ([T]s, **A**), cortisol ([C]s, **B**), alpha-amylase ([AA]s, **C**) immunoglobulin A ([IgA]s, **D**) and chromogranin A ([CgA]s, **E**), and plasma creatine kinase (CK) activity **(F)** during preparation (PREP) and pre-competitive (COMP) periods for male (M) and female (F) athletes. ^*^Significant effect of gender (*P* < 0.05). †Significant difference between PREP and COMP (*P* < 0.05). Values are presented as mean ± SD.

Salivary testosterone concentration during PREP was 405.5 ± 112.7 and 164.7 ± 60.9 pmol.L^−1^ for male and female athletes, respectively vs. 409.6 ± 76.9 and 205.9 ± 83.2 pmol.L^−1^ during COMP. We observed a main effect of gender (*P* < 0.0001), while no main effect of training period (*P* = 0.29) or gender × training period interaction (*P* = 0.38) were found for testosterone concentration (Figure 2A). However, the variations of testosterone during COMP showed a significant effect of time (*P* = 0.03), with a significant increase of salivary testosterone at week 5 in comparison to week 2 (+14.5 ± 44.7%), week 3 (+9.2 ± 51.4%), and week 4 (+8.9 ± 50.5%; *P* < 0.05; Table 3).

**Table 3:** **Salivary markers and plasma CK activity of track and field athletes throughout the 5 weeks of the pre-competitive (COMP) period of the sport season**.

**POMS subscale scores**	**Training week of the pre-competitive period (COMP)**				
	**1**	**2**	**3**	**4**	**5**
[Testosterone]s	287.2 (150.2)	262.5 (124.9)	282.4 (128.7)	265.6 (119.1)	328.9 (175.9)^*^
[Cortisol]s	5.1 (3.2)	4.6 (3.0)	4.6 (2.6)	4.9 (4.6)	6.1 (4.3)
[Alpha-amylase]s	109.0 (72.5)	116.6 (69.8)	139.2 (89.0)	112.4 (70.2)	125.2 (77.9)
[Immunoglobulin A]s	139.2 (46.3)	111.5 (57.2)	143.1 (71.6)	151.3 (97.0)	141.6 (68.7)
[Chromogranin A]s	9.4 (15.4)	4.4 (1.8)	8.5 (9.7)	7.9 (11.7)	9.7 (9.8)
Creatine kinase activity	386.8 (243.4)	310.1 (186.9)	381.1 (240.6)	297.1 (230.8)	279.1 (168.4)

*The results are means (SD)*.

* *Significant effect of time (P > 0.05)*.

#### Cortisol

Salivary cortisol concentration during PREP was 6.8 ± 4.4 and 4.6 ± 2.3 nmol.L^−1^ for male and female athletes, respectively vs. 4.1 ± 1.8 and 5.6 ± 3.7 nmol.L^−1^ during COMP. No main effect of gender (*P* = 0.76) or time (*P* = 0.15) were found for salivary cortisol. A gender × training period interaction was obtained on cortisol concentration (*P* < 0.007), whereas *post-hoc* did not show significant differences between the different time points (*P* > 0.05; Figure 2B). Detailed analysis of the amount of cortisol in saliva did not reveal any significant variation throughout COMP (*P* = 0.64; Table 3). The T/C ratio (× 10^−3^) was higher in COMP compared to PREP (58.5 ± 39.9 vs. 80.9 ± 44.3; *P* = 0.01).

#### AA

Salivary AA concentration during PREP was 112.6 ± 50.7 and 106.7 ± 98.3 nmol.L^−1^ for male and female athletes, respectively vs. 142.2 ± 82.7 and 109.8 ± 55.6 nmol.L^−1^ during COMP. No effect of gender (*P* = 0.58), training period (*P* = 0.30) or gender × training period (*P* = 0.40) were observed (Figure 2C). No significant difference was observed between the 5 consecutive weeks of COMP (*P* = 0.23; Table 3) in salivary AA activity.

#### IgA

Salivary IgA concentration during PREP was 104.5 ± 39.9 and 87.2 ± 42.0 nmol.L^−1^ for male and female athletes, respectively vs. 146.9 ± 65.7 and 134.8 ± 55.1 nmol.L^−1^ during COMP. No main effect of gender (*P* = 0.43) was observed, whereas a significant effect of training period (*P* = 0.006) was obtained on salivary IgA, with no gender × training period interaction (*P* = 0.86). On average, salivary IgA levels measured in saliva of the elite athletes showed a significant increase between PREP and COMP (+60.5 ± 80.3%; *P* = 0.003; Figure 2D). No significant variation of salivary IgA was observed throughout COMP (*P* = 0.22; Table 3).

#### CgA

Salivary CgA concentration during PREP was 18.8 ± 14.4 and 13.6 ± 14.3 nmol.L^−1^ for male and female athletes, respectively vs. 13.6 ± 14.3 and 7.1 ± 5.0 nmol.L^−1^ during COMP. No main effect of gender was observed on salivary CgA (*P* = 0.37). A significant main effect of training period was obtained (*P* = 0.04), with no gender × training period interaction (*P* = 0.93). On average, CgA concentration decreased by 27.0 ± 82.8% from PREP to COMP (*P* = 0.005; Figure 2E). Week-by-week results did not show any main effect of time during the COMP period (*P* = 0.69; Table 3).

### CK activity

We observed a main effect of gender (*P* < 0.0001), training period (*P* < 0.0001) and a gender × training period interaction for plasma CK activity (*P* = 0.006). On average, CK activity significantly increased from 151.4 ± 35.9 to 448.4 ± 179.6 U.L^−1^ between PREP and COMP for male (*P* = 0.001) with no significant changes for female athletes (*P* = 0.48; Figure 2F). No effect of time was observed throughout COMP for CK activity (*P* = 0.09; Table 3).

### Correlation with training load

None of the psychological or physiological parameters were significantly correlated to the amount of training load during COMP (*P* > 0.05).

## Discussion

The present study aimed to compare the endocrine, psychological, and muscle damage response of world-class level athletes in preparation and pre-competitive period, where the injury incidence has been shown to increase. On the one hand, according to our hypothesis we observed changes in stress response manifested by a decrease in salivary chromogranin A concentration, and an immunological status improvement as shown by the increase in salivary IgA. On the other hand, the diminution of the psychological component of fatigue and training load during the pre-competitive period was not accompanied by significant variations in testosterone and cortisol responses. However, elite athletes exhibited higher CK activity during the pre-competitive period compared to the preparation period. This interesting result could potentially mirror the effect of high-intensity and specialized training, which could exacerbate the exercise-induced muscle damage resulting from this training cycle.

Due to the characteristics of the sample (international level track and field athletes), conditioning and training programs were determined entirely by the coaches. Although we were not allowed to define the training stimulus in this context, this study is the first to present the psycho-physiological response to long-term elite athletic training (4.5 months), under real conditions. According to the well-known classical models of training periodization, preparatory period programs contain extensive, high volume, and diversified exercises, whereas the competitive period is focused mainly on more intensified, specialized exercises of reduced volume (Issurin, 2010). Such a training design aims to lead the athletes to their seasonal peak performance at the moment of competition. Track and field athletics encompassed a wide variety of athletes with different training contents to reach the targeted performance level. The quantification of the workload resulting from such various training is noticeably complex. For this reason, the training load was measured using the calculation method proposed by Foster (1998), which provides a global evaluation of the exercise stimulus, applicable to the various activities. Based on this methodological approach, as expected, the training load progressively decreased as competition was approaching (Table 2). It must be kept in mind that varied training regimes are employed to prepare sprinters, middle distance runners, and combined events. Therefore, further investigations are required to specifically address the hormonal response to these respective physical loads.

This well-known reduction in training volume that precedes the competition was associated with a reduced fatigue perception as shown by the lower level of tension, depression, anger, confusion, and total mood disturbance in pre-competitive in comparison with the PREP (Table 1). Our findings are in accordance with previous studies that show a lower mood disturbance in low-load periods (e.g., recovery, taper) than in high-load periods (e.g., high-volume training) (Faude et al., 2008). Conversely, psychological alterations have been reported in athletes submitted to a training stress leading to an overreached state (Coutts et al., 2007). If the athletes' mood states were close to high-volume training in our study (Rietjens et al., 2005), they did not reach values observed in overreached subjects. This suggests that training contents are properly adjusted by the coaches to avoid chronic fatigue or overreaching at international competitive level, even during high-load programs, which are recognized as a potential cause of psychological disturbance.

The pre-competitive phase was identified as a season period conducive to an increased injury incidence in track and field athletes (D'souza, 1994). Such interruption in the training process before competition could be related to an imbalance between training stimulus and subsequent recovery (Foster, 1998). Overreached athletes have been shown to exhibit an exhaustion of the HPA axis (Meeusen et al., 2006). Although such influence on the higher brain centers can be inferred from changes in blood catecholamines, stress response was appraised based on hormonal variations in saliva. This easy-to-sample fluid allowed for non-invasive and stress-free determination of the physiological status of elite athletes with important time constraints. In addition, studies have shown that hormonal changes in response to exercise are more pronounced in saliva compared to blood (Gozansky et al., 2005). Given that steroids concentration may exhibit large fluctuations (Gatti and De Palo, 2011), we collected multiple samples to obtain relevant data. Saliva was collected in the middle of the afternoon when hormones concentrations exhibit reduced fluctuations in comparison with morning measurements, which could be influenced by the awakening response.

The present group of world-class level athletes presented slightly higher rates of salivary testosterone when compared to elite male (286.5 pg.mL^−1^; Kivlighan and Granger, 2006) and female athletes (87 pg.mL^−1^; Cook et al., 2012). As salivary testosterone has been reported to be related to the training level (Cook et al., 2012), our values could thus reflect the very high training level of the present group of athletes. Such higher testosterone baseline could indicate a greater capacity for performance at higher work rates. Hormones with anabolic or catabolic properties like testosterone show quantitative changes, reflective of a catabolic state, in relation to the intensity and duration of training load (Gatti and De Palo, 2011). Indeed, a negative relationship between training load and resting testosterone has been reported (Elloumi et al., 2008). Although training load and psychological perception of fatigue were lower in the pre-competitive period than in the preparation period in our study, testosterone concentration was similar in both phases (Figure 2A). This lack of variation between preparation and pre-competitive periods could be interpreted as a compensatory process between the load diminution (which potentially increases [T]s) associated with the reduction in strength training contents (which potentially decreases [T]s) (Gatti and De Palo, 2011).

Salivary cortisol is also recommended and extensively used as an index of training stress (Gatti and De Palo, 2011). The present cortisol values are in the range obtained for athletes (1.8–19.9 nmol.L^−1^; Cook et al., 2013), and close to the lowest concentrations commonly reported in the literature. This could be explained by the fact that measurements were performed in the middle of the afternoon (3–4 p.m.) when cortisol reaches a low-level plateau (Strahler et al., 2010). Moreover, the rise in cortisol induced by a psychobiological stress (i.e., training) has been demonstrated to be lower in trained than in untrained subjects (Rimmele et al., 2007), which is confirmed by our results obtained on highly trained athletes. A robust rise in resting cortisol has been reported as a complementary approach to assess excessive fatigue at the onset of overreaching (Urhausen et al., 1998). However, as for testosterone, we did not observe significant changes in cortisol concentration between preparation and pre-competitive periods. Only the T/C ratio was increased in pre-competitive period, which could reflect the lower amount of training load during this training cycle. Our findings thus suggest a similar response of HPA axis to high-load or high-intensity training in track and field elite athletes. Such a result could also originate from the expertise of the coaches and the athletes themselves in their aptitude to properly regulate the training load according to the athletes' capacities, as reported for elite subjects (Cook and Beaven, 2013).

However, stress is a multi-faceted phenomenon that requires a multidimensional measurements approach (Nater et al., 2006). Indeed, except HPA axis, exercise-induced stress is also mediated by the ANS, which stimulates the adrenal medulla to produce catecholamines. Intense training (e.g., pre-competitive period) can deplete the amount of catecholamines in the blood. As AA and CgA are co-released with catecholamines in saliva, and these hormones have been proposed as non-invasive markers of autonomic activity. Although previous data on elite athletes are scarce, our study showed similar AA concentrations as those obtained by Filaire et al. (2013) on tennis players (125 U.mL^−1^). CgA values also showed salivary concentrations close to active subjects (8.9 pg.mL-1; Gallina et al., 2011). Competition involves social comparison and evaluation, which represents a potential source of pressure for athletes. Competitive periods are thus recognized for increasing sympathetic activity both in the anticipation and in the response to the event, which results in significant increase in salivary AA and CgA (Kraemer et al., 2001). While salivary AA increases might reflect the interaction of stress-dependent sympathetic and parasympathetic stimulation via central noradrenergic input, AA response depends on the nature of the stressor (Nater and Rohleder, 2009). Consequently, the changes in training contents between preparation and pre-competitive phases could have different effects on the AA response in the present group of elite athletes. This hypothesis was not verified, because similar concentrations were measured in both periods (Figure 2C). Inversely, CgA concentration was significantly decreased during the pre-competitive phase. Even though the mechanisms underlying its salivary secretion are complex, it is suggested that CgA is involved in the secretory responses to α-adrenergic agonists and is therefore considered a reliable index of autonomic activity (Gallina et al., 2011). Most of the studies based on salivary CgA were interested in the acute response to exercise, whereas long-term studies are scarce. Our findings suggest that a reduced training load decreases the salivary release of CgA at rest, and this could potentially indicate a lower autonomic activity in pre-competitive phases for elite athletes (Montero-Hadjadje et al., 2008).

As immunoglobulins A act as the first line of defense via mucosal surface, the assessment of IgA represents the effects of exercise on mucosal immunity. While variable effects of acute exercise on IgA have been reported in the literature, chronic exercise (training) can decrease the salivary concentration of IgA (Libicz et al., 2006). Such a decline in IgA appears to contribute to increase the athletes' susceptibility for upper respiratory tract infection (Neville et al., 2008; Papacosta and Nassis, 2011). The present IgA concentrations existed in the variations reported of a training season of athletes (35–314 μg.mL^−1^; Neville et al., 2008), with significant higher values in pre-competitive compared to preparation period (Figure 2D). The reduction in training load as competitive events approach seemed to positively affect the mucosal immunity of elite athletes. Together with the reduced autonomic activity inferred from CgA results, these findings suggest that the rise in injury incidence during pre-competitive periods could not be attributed to an excessive general fatigue that could have triggered ANS disturbance or immunodepression.

Blood CK activity increased in pre-competitive period when compared with preparation period. Such increase in CK content is observed in the case of degeneration and regeneration of skeletal muscle damage (Guilhem et al., 2010). Although our CK values are comprised in the ranges reported throughout an athletic season (Meyer and Meister, 2011), the significant increase observed in pre-competitive period could be reflective of higher levels of muscle damage induced by intensified and specialized exercises, which increase the mechanical strain and the risk of injury in muscular tissues (Fiorentino et al., 2014). However, CK is recognized for being influenced by factors other than the level of muscle damage (e.g., soft tissue trauma, exercise-induced hemoconcentration and/or hemodilution, alterations of tissue clearance). Hence, the measurement of muscle injury cannot be solely based on the changes of CK activity (Guilhem et al., 2013). Furthermore, week-to-week analysis of salivary contents did not reveal significant changes in any of the assessed hormones (Table 3). Although ANS and HPA response could serve to identify overstressed athletes (i.e., functional or non-functional overreaching; Papacosta and Nassis, 2011), these data also illustrate the high inter- and intra-individual variability of hormonal response to exercise. Consequently, taken together with the lack of acute changes during pre-competitive period when injury incidence increases, our study suggests that potential increase in risk of injury from an increase in training load is less likely than from an increase in musculo-articular load. Innovative markers (e.g., micro-ARN; Banzet et al., 2013), more specific to tissue lesions resulting from high-intensity workouts, should be thus considered in power-oriented activities with repeated breaking (eccentric) actions, such as track and field athletics. Such indicators would benefit from optimization in the quantification procedure and increase in the capacity to perform large number of simultaneous measurements to allow for their implementation in high-level training conditions.

The present study aimed to determine the psychological, endocrinological, immunological, and muscle damage status throughout the season of world-class level track and field athletes, based on the concentration of hormones contained in saliva and plasma CK activity. The reduction in training load during pre-competitive period compared to preparation period was associated with a decrease in psychological fatigue. Although T/C ratio and IgA were increased, and CgA decreased in response to the reduction in training load and fatigue perception, these parameters were not correlated to the training load. These findings suggest that saliva composition is sensitive to the type of training workouts performed by track and field athletes during the various season periods. Nonetheless, salivary hormones could not be related to the amount of workload resulting from athletics training, particularly during pre-competitive training period where injury incidence has been reported to increase. Our findings suggest that innovative markers, more specific to muscle tissue lesions, should be considered in the future to diagnose muscular overuse. The implementation of such indicators would help coaches and athletes to optimize the regulation of training contents throughout the season, particularly when high-intensity specialized workouts are used to increase technical performance as competition approaches.

## Author contributions

Conception or design of the work: GG, CH, AG, MC. Acquisition, analysis, or interpretation of data for the work: GG, CH, NG, DB, AG, MC. Drafting the work or revising it critically for important intellectual content: GG, CH, NG, DB, AG, MC. Final approval of the version to be published: GG, CH, NG, DB, AG, MC. Agreement to be accountable for all aspects of the work in ensuring that questions related to the accuracy or integrity of any part of the work are appropriately investigated and resolved: GG, CH, NG, DB, AG, MC.

## Funding

The study was funded by the French Ministry of Sports (contract no. 10-i-008) and received grants from the French Athletics Federation.

### Conflict of interest statement

The authors declare that the research was conducted in the absence of any commercial or financial relationships that could be construed as a potential conflict of interest.

## References

[B1] Adlercreutz H. Härkönen M. Kuoppasalmi K. Näveri H. Huhtaniemi I. Tikkanen H.. (1986). Effect of training on plasma anabolic and catabolic steroid hormones and their response during physical exercise. Int. J. Sports Med. 7(Suppl. 1), 27–28. 10.1055/s-2008-1025798 3744643

[B2] Banzet S. Chennaoui M. Girard O. Racinais S. Drogou C. Chalabi H.. (2013). Changes in circulating microRNAs levels with exercise modality. J. Appl. Physiol. 115, 1237–1244. 10.1152/japplphysiol.00075.2013 23950168

[B3] Born D. P. Faiss R. Willis S. J. Strahler J. Millet G. P. Holmberg H. C.. (2015). Circadian variation of salivary immunoglobin A, alpha-amylase activity and mood in response to repeated double-poling sprints in hypoxia. Eur. J. Appl. Physiol.. [Epub ahead of print]. 10.1007/s00421-015-3236-3 26269448

[B4] Cayrou S. Dickès P. Dolbeault S. (2003). [French version of the Profile of Mood State (POMS-f)]. Sci. Sport 13, 83–88.

[B5] Chennaoui M. Gomez-Marino D. Drogou C. Bourrilhon C. Sautivet S. Guezennec C. Y. (2004). Hormonal and metabolic adaptation in professional cyclists during training. Can. J. Appl. Physiol. 29, 714–730. 10.1139/h04-046 15630145

[B6] Cook C. J. Beaven C. M. (2013). Salivary testosterone is related to self-selected training load in elite female athletes. Physiol. Behav. 116–117, 8–12. 10.1016/j.physbeh.2013.03.013 23531473

[B7] Cook C. J. Crewther B. T. Kilduff L. P. (2013). Are free testosterone and cortisol concentrations associated with training motivation in elite male athletes? Psychol. Sport Exerc. 14, 882–885. 10.1016/j.psychsport.2013.08.001

[B8] Cook C. J. Crewther B. T. Smith A. A. (2012). Comparison of baseline free testosterone and cortisol concentrations between elite and non-elite female athletes. Am. J. Hum. Biol. 24, 856–858. 10.1002/ajhb.22302 22915557

[B9] Coutts A. J. Wallace L. K. Slattery K. M. (2007). Monitoring changes in performance, physiology, biochemistry, and psychology during overreaching and recovery in triathletes. Int. J. Sports Med. 28, 125–134. 10.1055/s-2006-924146 16835823

[B10] Díaz Gómez M. M. Bocanegra Jaramillo O. L. Teixeira R. R. Espindola F. S. (2013). Salivary surrogates of plasma nitrite and catecholamines during a 21-week training season in swimmers. PLoS ONE 8:e64043. 10.1371/journal.pone.0064043 23700456 PMC3660304

[B11] Diaz M. M. Bocanegra O. L. Teixeira R. R. Soares S. S. Espindola F. S. (2013). Salivary nitric oxide and alpha-amylase as indexes of training intensity and load. Int. J. Sports Med. 34, 8–13. 10.1055/s-0032-1316318 22960992

[B12] D'souza D. (1994). Track and field athletics injuries—a one-year survey. Br. J. Sports Med. 28, 197–202. 10.1136/bjsm.28.3.197 8000821 PMC1332067

[B13] Elloumi M. Ben Ounis O. Tabka Z. Van Praagh E. Michaux O. Lac G. (2008). Psychoendocrine and physical performance responses in male Tunisian rugby players during an international competitive season. Aggress. Behav. 34, 623–632. 10.1002/ab.20276 18626966

[B14] Faude O. Meyer T. Scharhag J. Weins F. Urhausen A. Kindermann W. (2008). Volume vs. intensity in the training of competitive swimmers. Int. J. Sports Med. 29, 906–912. 10.1055/s-2008-1038377 18418808

[B15] Filaire E. Ferreira J. P. Oliveira M. Massart A. (2013). Diurnal patterns of salivary alpha-amylase and cortisol secretion in female adolescent tennis players after 16 weeks of training. Psychoneuroendocrinology 38, 1122–1132. 10.1016/j.psyneuen.2012.11.001 23200107

[B16] Fiorentino N. M. Rehorn M. R. Chumanov E. S. Thelen D. G. Blemker S. S. (2014). Computational models predict larger muscle tissue strains at faster sprinting speeds. Med. Sci. Sports Exerc. 46, 776–786. 10.1249/MSS.0000000000000172 24145724 PMC3960352

[B17] Foster C. (1998). Monitoring training in athletes with reference to overtraining syndrome. Med. Sci. Sports Exerc. 30, 1164–1168. 10.1097/00005768-199807000-00023 9662690

[B18] Gallina S. Di Mauro M. D'Amico M. A. D'Angelo E. Sablone A. Di Fonso A.. (2011). Salivary chromogranin A, but not alpha-amylase, correlates with cardiovascular parameters during high-intensity exercise. Clin. Endocrinol. (Oxf.) 75, 747–752. 10.1111/j.1365-2265.2011.04143.x 21671973

[B19] Gatti R. De Palo E. F. (2011). An update: salivary hormones and physical exercise. Scand. J. Med. Sci. Sports 21, 157–169. 10.1111/j.1600-0838.2010.01252.x 21129038

[B20] Gozansky W. S. Lynn J. S. Laudenslager M. L. Kohrt W. M. (2005). Salivary cortisol determined by enzyme immunoassay is preferable to serum total cortisol for assessment of dynamic hypothalamic—pituitary—adrenal axis activity. Clin. Endocrinol. (Oxf.) 63, 336–341. 10.1111/j.1365-2265.2005.02349.x 16117823

[B21] Granger D. A. Weisz J. R. McCracken J. T. Kauneckis D. Ikeda S. (1994). Testosterone and conduct problems. J. Am. Acad. Child Adolesc. Psychiatry 33, 908. 10.1097/00004583-199407000-00020 8083150

[B22] Guilhem G. Cornu C. Guével A. (2010). Neuromuscular and muscle-tendon system adaptations to isotonic and isokinetic eccentric exercise. Ann. Phys. Rehabil. Med. 53, 319–341. 10.1016/j.rehab.2010.04.003 20542752

[B23] Guilhem G. Hug F. Couturier A. Regnault S. Bournat L. Filliard J. R.. (2013). Effects of air-pulsed cryotherapy on neuromuscular recovery subsequent to exercise-induced muscle damage. Am. J. Sports Med. 41, 1942–1951. 10.1177/0363546513490648 23739686

[B24] Issurin V. B. (2010). New horizons for the methodology and physiology of training periodization. Sports Med. 40, 189–206. 10.2165/11319770-000000000-00000 20199119

[B25] Kivlighan K. T. Granger D. A. (2006). Salivary alpha-amylase response to competition: relation to gender, previous experience, and attitudes. Psychoneuroendocrinology 31, 703–714. 10.1016/j.psyneuen.2006.01.007 16624493

[B26] Kraemer W. J. Fry A. C. Rubin M. R. Triplett-McBride T. Gordon S. E. Koziris L. P.. (2001). Physiological and performance responses to tournament wrestling. Med. Sci. Sports Exerc. 33, 1367–1378. 10.1097/00005768-200108000-00019 11474340

[B27] Le Meur Y. Hausswirth C. Natta F. Couturier A. Bignet F. Vidal P. P. (2013). A multidisciplinary approach to overreaching detection in endurance trained athletes. J. Appl. Physiol. 114, 411–420. 10.1152/japplphysiol.01254.2012 23195630

[B28] Libicz S. Mercier B. Bigou N. Le Gallais D. Castex F. (2006). Salivary IgA response of triathletes participating in the French Iron Tour. Int. J. Sports Med. 27, 389–394. 10.1055/s-2005-865747 16729381

[B29] Meeusen R. Duclos M. Gleeson M. Rietjens G. Steinacker J. Urhausen A. (2006). Prevention, diagnosis and treatment of the Overtraining Syndrome. Eur. J. Sport Sci. 6, 1–14. 10.1080/17461390600617717 23247672

[B30] Meyer T. Meister S. (2011). Routine blood parameters in elite soccer players. Int. J. Sports Med. 32, 875–881. 10.1055/s-0031-1280776 22020850

[B31] Montero-Hadjadje M. Vaingankar S. Elias S. Tostivint H. Mahata S. K. Anouar Y. (2008). Chromogranins A and B and secretogranin II: evolutionary and functional aspects. Acta Physiol. 192, 309–324. 10.1111/j.1748-1716.2007.01806.x 18005393

[B32] Nater U. M. La Marca R. Florin L. Moses A. Langhans W. Koller M. M.. (2006). Stress-induced changes in human salivary alpha-amylase activity—associations with adrenergic activity. Psychoneuroendocrinology 31, 49–58. 10.1016/j.psyneuen.2005.05.010 16002223

[B33] Nater U. M. Rohleder N. (2009). Salivary alpha-amylase as a non-invasive biomarker for the sympathetic nervous system: current state of research. Psychoneuroendocrinology 34, 486–496. 10.1016/j.psyneuen.2009.01.014 19249160

[B34] Neville V. Gleeson M. Folland J. P. (2008). Salivary IgA as a risk factor for upper respiratory infections in elite professional athletes. Med. Sci. Sports Exerc. 40, 1228–1236. 10.1249/MSS.0b013e31816be9c3 18580401

[B35] Papacosta E. Nassis G. P. (2011). Saliva as a tool for monitoring steroid, peptide and immune markers in sport and exercise science. J. Sci. Med. Sport 14, 424–434. 10.1016/j.jsams.2011.03.004 21474377

[B36] Rietjens G. J. Kuipers H. Adam J. J. Saris W. H. van Breda E. van Hamont D.. (2005). Physiological, biochemical and psychological markers of strenuous training-induced fatigue. Int. J. Sports Med. 26, 16–26. 10.1055/s-2004-817914 15643530

[B37] Rimmele U. Zellweger B. C. Marti B. Seiler R. Mohiyeddini C. Ehlert U.. (2007). Trained men show lower cortisol, heart rate and psychological responses to psychosocial stress compared with untrained men. Psychoneuroendocrinology 32, 627–635. 10.1016/j.psyneuen.2007.04.005 17560731

[B38] Strahler J. Berndt C. Kirschbaum C. Rohleder N. (2010). Aging diurnal rhythms and chronic stress: distinct alteration of diurnal rhythmicity of salivary alpha-amylase and cortisol. Biol. Psychol. 84, 248–256. 10.1016/j.biopsycho.2010.01.019 20138206

[B39] Tsigos C. Chrousos G. P. (2002). Hypothalamic-pituitary-adrenal axis, neuroendocrine factors and stress. J. Psychosom. Res. 53, 865–871. 10.1016/S0022-3999(02)00429-4 12377295

[B40] Urhausen A. Gabriel H. Kindermann W. (1995). Blood hormones as markers of training stress and overtraining. Sports Med. 20, 251–276. 10.2165/00007256-199520040-00004 8584849

[B41] Urhausen A. Gabriel H. H. Kindermann W. (1998). Impaired pituitary hormonal response to exhaustive exercise in overtrained endurance athletes. Med. Sci. Sports Exerc. 30, 407–414. 10.1097/00005768-199803000-00011 9526887

[B42] Wood P. (2009). Salivary steroid assays - research or routine? Ann. Clin. Biochem. 46, 183–196. 10.1258/acb.2008.008208 19176642

